# Therapeutic targeting and rapid mobilization of endosteal HSC using a small molecule integrin antagonist

**DOI:** 10.1038/ncomms11007

**Published:** 2016-03-15

**Authors:** Benjamin Cao, Zhen Zhang, Jochen Grassinger, Brenda Williams, Chad K. Heazlewood, Quentin I. Churches, Simon A. James, Songhui Li, Thalia Papayannopoulou, Susan K. Nilsson

**Affiliations:** 1 Biomedical Manufacturing, CSIRO Manufacturing, Bag 10, Clayton South, Victoria 3169, Australia; 2 Australian Regenerative Medicine Institute, Monash University, Clayton, Victoria 3800, Australia; 3 University Hospital Regensberg, Department of Hematology and Oncology, Franz-Josef-Strauß-Allee 11, Regensburg 93053, Germany; 4 Australian Synchrotron, Clayton, Victoria 3168, Australia; 5 Department of Medicine/Hematology, University of Washington Seattle, 1705 NE Pacific, Box 357710, Seattle, Washington 98195-7710, USA

## Abstract

The inherent disadvantages of using granulocyte colony-stimulating factor (G-CSF) for hematopoietic stem cell (HSC) mobilization have driven efforts to identify alternate strategies based on single doses of small molecules. Here, we show targeting α_9_β_1_/α_4_β_1_ integrins with a single dose of a small molecule antagonist (BOP (*N*-(benzenesulfonyl)-L-prolyl-L-*O*-(1-pyrrolidinylcarbonyl)tyrosine)) rapidly mobilizes long-term multi-lineage reconstituting HSC. Synergistic engraftment augmentation is observed when BOP is co-administered with AMD3100. Impressively, HSC in equal volumes of peripheral blood (PB) mobilized with this combination effectively out-competes PB mobilized with G-CSF. The enhanced mobilization observed using BOP and AMD3100 is recapitulated in a humanized NODSCIDIL2Rγ^−/−^ model, demonstrated by a significant increase in PB CD34^+^ cells. Using a related fluorescent analogue of BOP (R-BC154), we show that this class of antagonists preferentially bind human and mouse HSC and progenitors via endogenously primed/activated α_9_β_1_/α_4_β_1_ within the endosteal niche. These results support using dual α_9_β_1_/α_4_β_1_ inhibitors as effective, rapid and transient mobilization agents with promising clinical applications.

## Abstract (web summary)

Mobilizing haematopoietic stem cells to the peripheral blood has largely replaced bone marrow transplants as a strategy in the clinic. Here, Cao *et al.* report the use of an α_9_β_1_/α_4_β_1_ integrin antagonist to induce rapid mobilization of blood stem cells from the bone marrow in a humanized mouse model.

## Graphical abstract

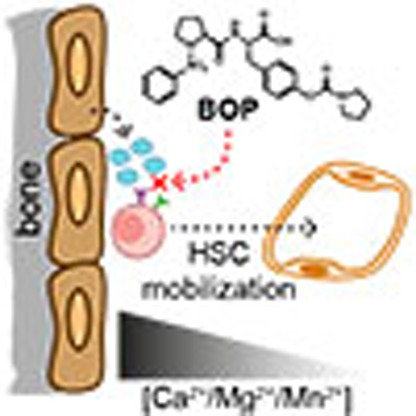

The transplant of mobilized peripheral blood (PB) hematopoietic stem cell (HSC) into patients undergoing treatment for blood diseases has essentially replaced traditional bone marrow (BM) transplants. Current clinical practice for HSC mobilization is achieved with an extended course of recombinant granulocyte colony-stimulating factor (G-CSF), which stimulates the production of proteases that cleave many interactions including CXCR4/SDF-1 (refs 1, 2). However, G-CSF-based mobilization requires multiple doses over a number of days, is known to alter the function of the HSC niche as well as bone formation^3^, can cause bone pain and spleen enlargement and on rare occasions G-CSF results in splenic rupture, myocardial infarction or cerebral ischaemia (reviewed in refs 4, 5). These inherent disadvantages of G-CSF have driven efforts to identify alternate mobilization strategies based on the use of single doses of small molecules^6^,^7^. For example, the Food and Drug Administration (FDA)-approved chemokine (C-X-C motif) receptor 4 (CXCR4) antagonist AMD3100 (Plerixafor; Mozobil^TM^) has been shown to rapidly mobilize HSC with limited toxicity issues^8^,^9^. Nevertheless, clinical mobilization with AMD3100 is only effective in combination with G-CSF and the search for rapid, selective and G-CSF-independent mobilization regimes remains a topic of interest.

HSC regulation and retention within the BM stem cell niche is mediated through multiple interactions between HSC surface receptors and their respective ligands expressed or secreted by surrounding cells: including osteoblasts, sinusoidal endothelial cells and perivascular cells (reviewed in refs 10, 11, 12). Functional analysis of BM HSC demonstrate those with the greatest hematopoietic potential, which preferentially localize in the endosteal region (∼12 cell diameters from the bone surface^13^). Of note, HSC identical for the classic Lin^−^Sca-1^+^ckit^+^CD150^+^CD48^−^ phenotype, but isolated from endosteal BM have greater homing potential and enhanced long-term, multi-lineage hematopoietic reconstitution relative to HSC isolated from the central medullary cavity^14^. Thus, the therapeutic targeting of endosteal HSC for mobilization should provide better transplant outcomes.

Both α_4_β_1_ (VLA-4) and α_9_β_1_ expressed by HSC have been implicated in niche retention through binding to thrombin-cleaved osteopontin (trOpn) in the endosteal region^15^,^16^ and vascular cell adhesion molecule 1 (VCAM-1) expressed by BM stromal cells^17^. While the role of α_9_β_1_ in HSC mobilization is unknown, the down regulation of Opn using non-steroidal anti-inflammatory drugs as well as targeting α_4_ (for example, α_4_β_1_ antagonist BIO5192) with or without AMD3100 or G-CSF have validated trOpn/VCAM-1 binding to integrins as effective targets for HSC mobilization^6^,^18^,^19^,^20^. Nevertheless, the lack of suitable blocking antibodies and selective small molecules directed towards α_9_β_1_ has hindered our understanding of α_9_β_1_-dependent HSC mobilization. Furthermore, the specific cell types targeted for mobilization, as well as their location in BM have not previously been investigated.

We recently developed a fluorescent integrin antagonist, R-BC154 (ref. 21) based on the small molecule *N*-(benzenesulfonyl)-L-prolyl-L-*O*-(1-pyrrolidinylcarbonyl)tyrosine^22^ (BOP), which we previously demonstrated to bind effectively to α_9_β_1_ and α_4_β_1_ integrins in the presence of divalent metal cations^21^. Based on the interaction between α_9_β_1_/α_4_β_1_ and trOpn being restricted to endosteal BM, we postulated that this family of compounds would target potent endosteal HSC for mobilization. Herein, we show that R-BC154 and BOP preferentially bind endosteally located human and murine BM HSC and progenitors *in vitro* and *in vivo* and this binding is highly dependent on α_9_β_1_. Consequently, administration of a single dose of BOP results in the rapid and preferential mobilization of HSC and progenitors in mice and humanized mouse models, and is synergistically augmented when used in combination with AMD3100. When BOP is co-administered with AMD3100, greater mobilization is observed relative to AMD3100 in combination with the selective α_4_β_1_ antagonist BIO5192, identifying an active role for α_9_β_1_ in small molecule-mediated mobilization. A single dose of BOP and AMD3100 is also shown to mobilize HSC with greater long-term repopulation potential than HSC following a 4-day G-CSF regime. Together, our results show therapeutic targeting of BM HSC using α_9_β_1_/α_4_β_1_ inhibitors either alone or in combination with AMD3100 offers promising alternatives to current mobilization strategies.

## Results

### R-BC154 and BIO5192 reveals specific binding to HSC α_9_β_1_

**Figure 1:**
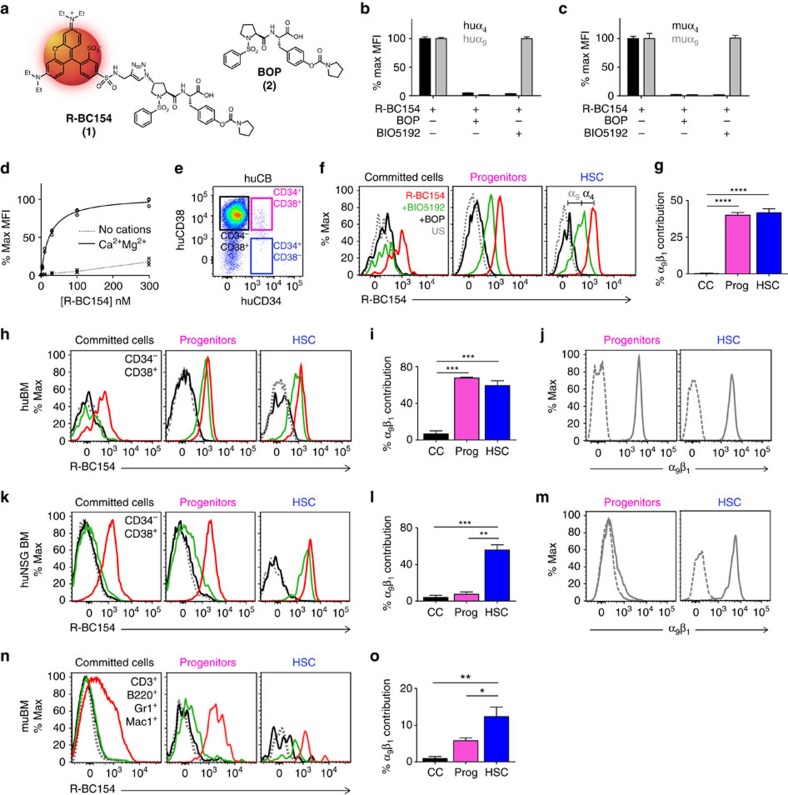
R-BC154 and BOP preferentially bind human and murine HSC and progenitors in a divalent cation and integrin-dependent manner. (**a**) Chemical structure of R-BC154 (1) and BOP (2). (**b**) Use of R-BC154, BOP and BIO5192 to demonstrate specific detection of binding to α_9_ (grey) in human and (**c**) murine overexpressing cell lines (LN18 and CHO respectively) compared with α_4_ (black) in the presence of 1 mM Ca^2+^/Mg^2+^ *n*=3 and is representative of at least two independent experiments. (**d**) Dose response of R-BC154 binding to human CB MNC. *n*=3 and is representative of two independent experiments. Binding in the presence of cations is significantly greater at all concentrations except 0 nM (*P*<0.005). (**e**) Representative populations of CB HSC (CD34^+^CD38^−^), progenitors (CD34^+^CD38^+^) and committed cells (CD34^−^CD38^+^). (**f**) R-BC154 binding to CB MNC committed cells, progenitors and HSC in the presence of 1 mM Ca^2+^/Mg^2+^ alone, or in combination with BOP or BIO5192. Data are representative of three individual samples. US, unstained. α_9_—specific binding via α_9_β_1_, α_4_—specific binding via α_4_β_1_. (**g**) Specific binding of R-BC154 binding to α_9_β_1_ on CB-committed cells (CC), progenitors (prog) and HSC. *n*=3 (**h**) R-BC154 binding to human BM (huBM). Data are representative of three individual samples. (**i**) Specific binding of R-BC154 to huBM α_9_β_1_. *n*=3 (**j**) α_9_β_1_ expression on huBM progenitors and HSC (grey solid line, dashed line IgG1 isotype control). Data are representative of three individual samples. (**k**) R-BC154 binding to humanized NSG (huNSG) BM. Data are representative of three individual samples. (**l**) Specific binding of R-BC154 to huNSG BM α_9_β_1_. *n*=3. (**m**) α_9_β_1_ expression on huNSG BM progenitors and HSC (grey solid line, dashed line IgG1 isotype control). Data are representative of three individual samples. (**n**) R-BC154 binding to mu BM-committed cells, progenitors (Lin^−^Sca-1^+^c-kit^+^; LSK) and HSC (LSKCD150^+^CD48^−^; LSKSLAM). Data are representative of three individual samples. (**o**) Specific binding of R-BC154 to mu BM α_9_β_1_. *n*=3. CB CD34^+^ cells were transplanted into 6.5 week old male (*n*=1) and female (*n*=2) NSG mice (**k**,**l**). Around 7–8-week-old female C57 mice were used for **n** and **o**. All data are mean±s.e.m. Analysis using one-way ANOVA (**g**,**i**,**l**,**o**) or two-way ANOVA (**d**), *P<0.05, **P<0.01, ***P<0.005, ****P<0.001.

To assess the specific targeting of α_9_β_1_ on HSC by small molecule antagonists, we developed an *in vitro* assay using the selective and potent (*K*_d_<10 pM) α_4_β_1_ antagonist BIO5192 (ref. 23), the dual α_9_β_1_/α_4_β_1_ antagonist BOP and its fluorescent analogue R-BC154 (Fig. 1a); which we have demonstrated to efficiently bind to both human^21^ and murine (Supplementary Fig. 1a) α_9_β_1_ and α_4_β_1_ integrins in a divalent metal cation-dependent manner. This is in contrast to BIO5192, which binds α_4_β_1_ in the presence and absence of divalent metal cations^23^. The co-labelling of both human and murine α_9_β_1_/α_4_β_1_ with R-BC154 and excess BIO5192 allowed the specific detection of binding to α_9_β_1_ (Fig. 1b,c). In contrast, R-BC154 labelling in combination with excess BOP completely inhibited R-BC154 binding to both human and murine α_9_β_1_/α_4_β_1_ integrins (Fig. 1b,c). Given the ubiquitous expression of α_4_β_1_ on all hematopoietic cells, the combination of R-BC154 with BIO5192 provides a convenient method of measuring specific binding to α_9_β_1_ on hematopoietic stem and progenitors.

### R-BC154 and BOP preferentially bind HSC and progenitors

We have previously demonstrated human HSC express α_9_β_1_ and its interaction with trOpn regulates HSC quiescence^16^. To determine whether dual α_9_β_1_/α_4_β_1_ antagonists bind human HSC via α_9_β_1_, R-BC154 binding to cord blood (CB) mononuclear cells (MNC) was assessed and shown to be divalent cation and dose dependent, and saturable (Fig. 1d). Analysis of CB HSC (CD34^+^CD38^−^), progenitors (CD34^+^CD38^+^) and lineage-committed cells (CD34^−^CD38^+^) (Fig. 1e) revealed high R-BC154 binding to HSC and progenitors, but only modest binding to committed cells in the presence of 1 mM Ca^2+^/Mg^2+^ (Fig. 1f). Furthermore, co-incubation of these populations with R-BC154 in combination with BIO5192 demonstrated partial inhibition of R-BC154 binding to HSC and progenitors (reflective of binding via both α_9_β_1_ and α_4_β_1_) and complete inhibition of binding to lineage-committed cells (reflective of binding only via α_4_β_1_) (Fig. 1f). In contrast, as BOP effectively binds both α_9_β_1_ and α_4_β_1_, its addition resulted in complete inhibition of R-BC154 binding to all cell populations (Fig. 1f). Together, these data demonstrate a significant proportion of R-BC154 binding to HSC and progenitors via α_9_β_1_, while binding to committed cells is mediated exclusively through α_4_β_1_ (Fig. 1g).

In addition, R-BC154 efficiently bound HSC and progenitors isolated from human BM in the presence of Ca^2+^/Mg^2+^(Fig. 1h) and similarly to that evident with CB cells, the majority of this binding was via α_9_β_1_ compared with committed cells that primarily bound via α_4_β_1_ (Fig. 1i). These data are consistent with the high α_9_β_1_ expression evident on human BM HSC and progenitors (Fig. 1j).

R-BC154 binding to human HSC was further evaluated using humanized NODSCIDIL2Rγ^−/−^ (huNSG) mice (Supplementary Fig. 1b)^24^. R-BC154-bound human BM-committed cells, progenitors and HSC in the presence of Ca^2+^/Mg^2+^ (Fig. 1k), but significant binding through α_9_β_1_ only occurred on HSC (Fig. 1l), which is consistent with α_9_β_1_ expression being restricted to HSC (Fig. 1m). In contrast, α_4_β_1_ was highly expressed on all three populations (Supplementary Fig. 1c).

The ubiquitous expression of α_4_β_1_ on hematopoietic cells, together with the restricted expression of α_9_β_1_ on HSC resulted in an additive R-BC154 binding to HSC relative to progenitors and committed cells (Supplementary Fig. 1d). Furthermore, single-cell correlation analysis of scaled fluorescence intensity values for both R-BC154 binding to α_9_β_1_ in the presence of BIO5192 (to prevent any binding to α_4_β_1_) and α_9_β_1_ expression (Supplementary Fig. 1e) shows a positive correlation in specific binding of BOP to α_9_β_1_ on CB HSC with the expression of α_9_β_1_. Together these data highlight the potential for human HSC and/or progenitors to be targeted in preference to lineage-committed cells using selective α_9_β_1_ antagonists.

Consistent with their human counterparts, analysis of R-BC154 binding to murine BM progenitors and HSC (Supplementary Fig. 1f; Fig. 1n) revealed this to be cation (Supplementary Fig. 1g) and α_4_/α_9_ (Supplementary Fig. 1h) dependent. Similarly to their human counterparts, a significant proportion of R-BC154 binding to murine HSC was via α_9_β_1_ whereas binding to lineage-committed cells was mediated primarily through α_4_β_1_ (Fig. 1o).

R-BC154 binding to murine BM progenitors and HSC was confirmed *in vivo* following subcutaneous administration of R-BC154, with similar levels of binding to that observed in the presence of exogenous Ca^2+^/Mg^2+^ *in vitro* (Supplementary Fig. 1i). The specific and reversible nature of R-BC154 binding *in vivo* was confirmed by efficient displacement of *in situ*-bound R-BC154 with the addition of excess competing BOP *in vitro* (Supplementary Fig. 1j,k).

### Endosteal HSC α_9_β_1_/α_4_β_1_ are endogenously activated

To date, a number of studies have demonstrated integrins adopt three conformational states: (1) inactive or low affinity; (2) activated (primed) or high affinity; and (3) ligand occupied (reviewed in ref. 25). This integrin activity is regulated via both inside-out activation or outside-in activation^26^,^27^. In the BM stem cell niche, the regulation of integrins on HSC is complex and has not been accurately mimicked or recapitulated *in vitro*^28^. In addition, we have previously shown that the region in which BM HSC reside has a profound influence on their function, with HSC isolated from the endosteal region having higher hematopoietic potential than their central counterparts^14^,^29^. Whilst the mechanisms of these differences remain poorly understood, significant differences in cellular and extracellular matrix molecules have been described in different BM regions, many of which are likely to play a role^30^,^31^.

**Figure 2:**
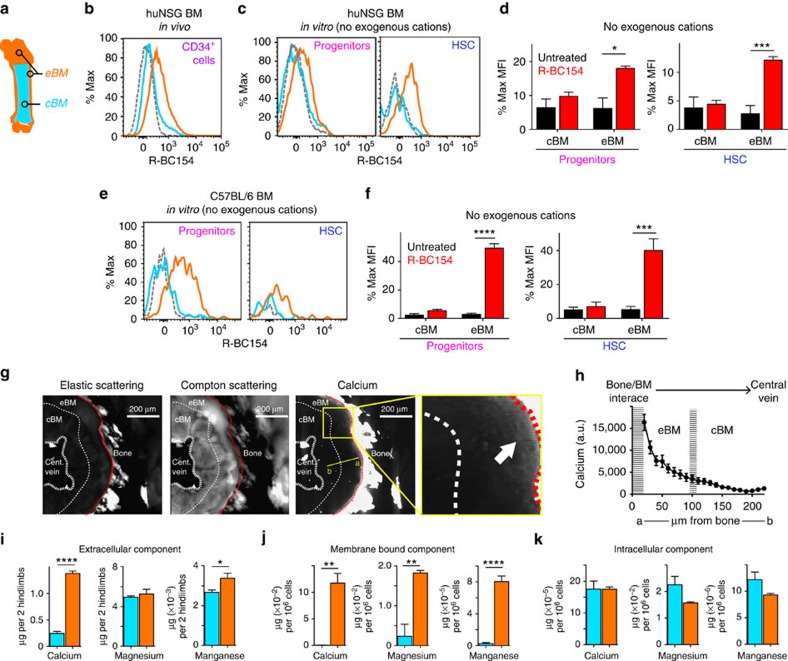
R-BC154 targets HSC and progenitors via endogenously activated/primed α_4_/α_9_ integrins in endosteal BM. (**a**) Schematic of central BM (cBM; blue) and endosteal BM (eBM; orange). (**b**) *in vivo* R-BC154 binding to huNSG central (blue) and endosteal (orange) BM huCD34^+^ cells compared with uninjected control (grey). Data are representative of three animals. (**c**) *In vitro* R-BC154 binding to huNSG central and endosteal BM progenitors (CD34^+^CD38^+^ cells) and HSC (CD34^+^CD38^−^ cells) in the absence of exogenous cations compared with uninjected control. Data are the representative of three samples. (**d**) Quantified R-BC154 binding from **c**, *n*=3. (**e**) *In vitro* R-BC154 binding to murine central and endosteal BM progenitors (LSK cells) and HSC (LSKSLAM cells) in the absence of exogenous cations compared to uninjected control. Data are representative of >10 samples. (**f**) Quantified R-BC154 binding from **e**, *n*=5. (**g**) Representative XFM images depicting the elastic scattering, Compton scattering and calcium distribution profile of a diaphyseal cross-section of non-decalcified murine femur. White arrow depicts calcium gradient radiating away from the endosteal surface (red dotted line). The hashed white line at ∼100 μm from the bone surface represents the interface between eBM and cBM. (**h**) Calcium content across representative line a to b in **g**, which begins ∼20 μm and extends to ∼220 μm from the endosteal surface. Data are expressed as arbitrary units (a.u.) *n*=10 different lines a to b. Representative of three animals. (**i**) Quantitative analysis of calcium, magnesium and manganese in murine BM central and endosteal extracellular fluid, (**j**) membrane bound (**k**) and intracellular. *n*=3. The endosteal extracellular component has been corrected for mechanically caused release (Supplementary Fig. 2a). CB CD34^+^ cells were transplanted into 9-week-old female NSG mice (**b**–**d**). Around 7–8-week-old female mice were used for **e**–**h**. All data are mean±s.e.m. Analysis using *t*-test, *P<0.05, ***P<0.005, ****P<0.001.

To determine whether α_9_β_1_/α_4_β_1_ integrins expressed by HSC and progenitors within different regions of BM are differentially primed/activated *in situ*, R-BC154 binding to endosteal and central human BM HSC in huNSG mice was assessed (Fig. 2a). Endosteal huCD34^+^ cells isolated from huNSG mice injected subcutaneously with R-BC154 exhibited significantly greater binding than their central counterparts (Fig. 2b). In addition, when huNSG BM cells from either endosteal or central regions were labelled with R-BC154 *in vitro* in the absence of exogenous cations, only binding to endosteal progenitors and HSC was detected (Fig. 2c,d) although no differences in the expression of α_9_β_1_ or α_4_β_1_ was evident on cells from the central or endosteal BM regions (Supplementary Fig. 1l). Similarly, R-BC154 binding in the absence of exogenous cations was also restricted to murine HSC and progenitors harvested from endosteal BM (Fig. 2e,f). These data demonstrate that integrins expressed by endosteal stem and progenitors are endogenously activated or primed and remain in this ligand-binding receptive conformational state post harvest.

### Divalent metal cations are concentrated at the endosteum

The precise mechanisms leading to integrin priming/activation *in vivo* remain unclear, although previous studies have demonstrated activation of α_4_ *in vitro* via a number of mechanisms including ligand binding^32^,^33^, divalent cations^34^ and cytokines^35^,^36^.

In the *in vivo* endosteal BM region, differences in α_9_β_1_/α_4_β_1_ activation states may in part be due to the binding of their ligands, such as trOpn, which we have previously demonstrated to be restricted to this region^16^. However, the endogenously primed integrin activation state may also be due to the higher concentrations of divalent metal cations (for example, Ca^2+^, Mg^2+^ and Mn^2+^) present in endosteal BM *in situ*. Elemental mapping of non-decalcified cross-sections of diaphyseal murine femoral BM using X-ray fluorescence microscopy (XFM) was used to assess calcium content. Areas of cortical bone and cells were verified by bright-field microscopy and could be distinguished by XFM based on their elastic, Compton and calcium scattering profiles. Specifically, bone is elastic^high^/Compton^high^/calcium^high^ while marrow cells are elastic^low^/Compton^high^/calcium^low^ (Fig. 2g). Measurement of calcium in BM demonstrated a distinct concentration gradient, emanating from the endosteal bone/BM interface and decreasing towards the central BM and central vein (Fig. 2g,h). XFM was unable to distinguish magnesium due to strong absorption of Mg K shell fluorescence by the air path and the intense Fe K shell fluorescence severely limited sensitivity to manganese.

In addition, BM magnesium, manganese and calcium content were assessed using inductively coupled plasma mass spectrometry (ICP-MS), which revealed a greater abundance of calcium and manganese in endosteal extracellular fluid relative to central BM (Fig. 2i) as well calcium, magnesium and manganese bound to the cellular membranes of endosteal cells relative to central BM cells (Fig. 2j). To ensure the accuracy of these measurements, the proportion of each cation that was mechanically released by grinding the bone to isolate the endosteal cells was measured and subtracted from the raw extracellular measurements (Supplementary Fig. 2a). Furthermore, the ability of the combination of these mechanically released cation concentrations to activate integrins on central BM cells was assessed by measuring the binding of R-BC154 to progenitors and HSC after treatment with the endosteal extracellular fluid (as assessed in Fig. 2i). As a further control, the measured concentrations of ‘mechanically released' cations were added to central marrow cells and their ability to activate integrins resulting in the binding of R-BC154 assessed. No evidence of R-BC154 was detected under any of these conditions (Supplementary Fig. 2b) confirming the endogenous activation of integrins before cell isolation.

In contrast, the concentrations of these three cations located intracellularly was equivalent in cells located in the endosteal and central BM regions (Fig. 2k). Collectively, these data demonstrate higher concentrations of divalent metal cations required for integrin function within endosteal BM. Together with extracellular matrix (ECM) ligands such as trOpn or VCAM-1 and other cytokines, this would result in integrins on endosteal HSC being maintained in an activated/primed/ligand-occupied conformational binding state, thus potentially forming the mechanism underpinning preferential targeting of endosteal HSC and progenitors by integrin antagonists such as R-BC154 and BOP.

### BOP rapidly and preferentially mobilizes HSC and progenitors

**Figure 3:**
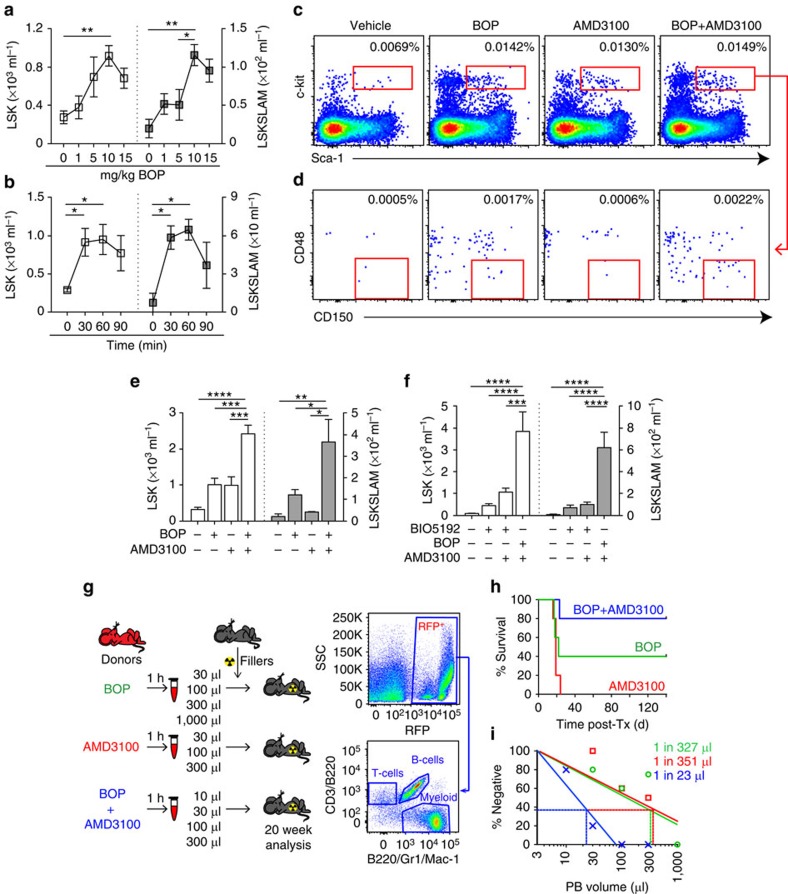
The α_9_β_1_/α_4_β_1_ integrin antagonist BOP rapidly mobilizes murine long-term repopulating HSC. (**a**) Dose-dependent mobilization of murine progenitors (LSK, white square) and HSC (LSKSLAM, grey square) with BOP. Data are pooled from two biological repeats; *n*>3 per group. (**b**) Time-course of progenitor and HSC mobilization with 10 mg kg^−1^ BOP. Time 0 is the vehicle control group. Data are pooled from two biological repeats. *n*=5 Individual animals per time point, not repetitive analysis. (**c**) Representative dot plots of PB progenitors and (**d**) HSC 1 h after a single dose of vehicle, BOP, AMD3100 or BOP+AMD3100 in combination, *n*>3. (**e**) Analysis of progenitor and HSC content in PB after 1 h treatment with vehicle, BOP, AMD3100 or BOP+AMD3100 in combination. (**f**) Total PB progenitor and HSC content 1 h after a single dose of vehicle (*n*=5), BIO5192 alone (*n*=5) or in combination with AMD3100 (*n*=5), or BOP in combination with AMD3100 (*n*=3). (**g**) Schematic of limiting dilution transplant analysis of mobilized RFP PB (five recipients per group). Representative dot plots of recipient BM multi-lineage engraftment. (**h**) Survival of recipients transplanted with 30 μl of RFP-mobilized PB. (**i**) Long-term HSC frequency in PB mobilized following BOP (green line), AMD3100 (red line) or BOP+AMD3100 (blue line). Around 7–8-week-old female (**a**–**e**) and male (**f**) C57 mice were used. 7–8 week old male (*n*=15) and female (*n*=10) RFP donors and male C57 recipients were used for **g**–**i**. All data are mean±s.e.m. Analysis using one-way ANOVA, *P<0.05, **P<0.01, ***P<0.005, ****P<0.001.

As BOP rapidly and preferentially binds BM HSC, the ability of BOP to mobilize HSC to the PB was initially analysed in a dose and time response assay and by quantifying progenitors (LSK) and HSC (LSKSLAM) post-subcutaneous BOP administration (Fig. 3a,b). The administration of BOP resulted in a rapid, significant and dose-dependent increase in PB progenitors and HSC (Fig. 3a), which peaked 60 min after a single dose (Fig. 3b) and returned to baseline within 4 h post administration (Supplementary Fig. 2c,d). Furthermore, whilst there was an initial significant increase in total PB lymphocytes, these also decreased to baseline by 18 h (Supplementary Fig. 2e).

In contrast, R-BC154, although capable of efficiently binding BM progenitors (LSK cells) and HSC (LSKSLAM cells) *in vitro* (Supplementary Fig. 1h), only resulted in moderate increase in PB progenitors and HSC when administered *in vivo* (Supplementary Fig. 2f). The reduced *in vivo* efficacy of R-BC154 relative to BOP is most likely the result of the former having lower binding affinities as determined by association and dissociation kinetics studies *in vitro*^21^,^22^ and thus reduced inhibitory potency towards integrin-dependent adhesive interactions.

### Using BOP and targeting CXCR4 enhances HSC mobilization

Previous studies have shown that the inhibition of α_4_β_1_ results in additive/synergistic HSC mobilization when used in combination with CXCR4 antagonists such as AMD3100 (refs 6, 37, 38). Administration of AMD3100 resulted in similar increase in WBC counts (7.8±1.5 × 10^6^ ml^−1^) relative to BOP (7.0±0.8 × 10^6^ ml^−1^) and comparable increases in the proportion of progenitors (LSK cells) in the PB (Fig. 3c). However, BOP resulted in a significantly greater increase in the proportion of HSC (LSKSLAM cells) in the PB relative to AMD3100 (Fig. 3d; *P*<0.05), suggesting AMD3100 predominantly mobilizes progenitors in addition to some HSC, while BOP predominantly mobilizes HSC in addition to some progenitors. In addition, the combination of BOP and AMD3100 synergistically mobilized WBC (16.4±1.9 × 10^6^ ml^−1^), progenitors and HSC compared with BOP or AMD3100 alone (Fig. 3e). Interestingly, significantly greater binding of BOP to endosteal LSK was observed relative to their central counterparts *in vivo* (Supplementary Fig. 2g), further supporting the data obtained using R-BC154 (Fig. 2b–f).

### Inhibiting α_9_β_1_, α_4_β_1_ and CXCR4 enhances HSC mobilization

To determine whether co-inhibition of α_9_β_1_ provides a mobilization advantage over the inhibition of α_4_β_1_ alone or in combination with CXCR4, the selective α_4_β_1_ inhibitor BIO5192 was used. BIO5192 has been reported to mobilize CFUs and long-term repopulating HSC with and without AMD3100 (ref. 6) but the specific cell types mobilized were not investigated. Intravenous administration of BIO5192 resulted in only moderate increase in WBC counts (Supplementary Fig. 2h), progenitors (LSK cells) and HSC (LSKSLAM cells) in the PB (Fig. 3f). Co-administration of BIO5192 with AMD3100 produced significant increases in total WBC (Supplementary Fig. 2h) but only a moderate 2.4-fold increase in progenitors and 1.4-fold increase in HSC compared with BIO5192 alone (Fig. 3f). This is in stark contrast to the combination of BOP and AMD3100, which mobilized significantly greater numbers of progenitors and HSC (Fig. 3f) despite inducing similar PB WBC counts (Supplementary Fig. 2h). These data demonstrate that concomitant binding to CXCR4 and α_4_β_1_ results in mobilization of predominantly committed WBC but in contrast, co-binding to α_4_β_1_ and α_9_β_1_ results in significantly increased HSC mobilization.

### Inhibiting α_9_β_1_ and α_4_β_1_ mobilizes functional HSC

To confirm whether the LSKSLAM and LSK cell phenotype in mobilized PB was reflective of functional HSC and progenitors with long-term multi-lineage engraftment potential, limiting dilution transplant analysis was performed using BOP, AMD3100 or the combination thereof (Fig. 3g). Greater survival was observed in recipients receiving 30 μl PB mobilized using the combination of BOP and AMD3100 compared with both BOP and AMD3100 alone (*P*<0.05) (Fig. 3h). Furthermore, Poisson regression analysis following limiting dilution transplant revealed PB mobilized by the BOP and AMD3100 combination resulted in a greater repopulation frequency of 1 HSC in 23 μl PB (95% confidence interval (CI)=1 HSC in 10 μl to 1 HSC in 51 μl) compared with BOP (1 HSC in 327 μl; 95% CI=1 HSC in 150 μl to 1 HSC in 715 μl) or AMD3100 (1 HSC in 351 μl; 95% CI=1 HSC in 128 μl to 1 HSC in 958 μl) alone, highlighting a greater than 10-fold improvement to mono-therapy (*P*<0.005) (Fig. 3i). These results demonstrate that treatment with BOP either alone or in combination with AMD3100, mobilizes long-term repopulating HSC and validates flow cytometric monitoring of the LSKSLAM phenotype as a rapid method for assessing murine HSC mobilization^39^.

### BOP+AMD3100 is an effective and rapid G-CSF alternative

**Figure 4:**
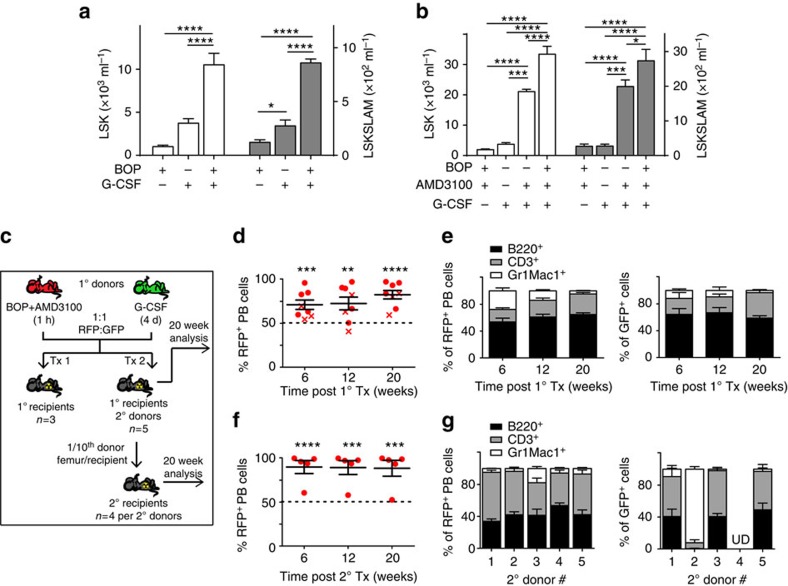
BOP and AMD3100 combination is a rapid and effective alternative to G-CSF. (**a**) Analysis of PB progenitor (LSK) and HSC (LSKSLAM) content following a single dose of BOP (*n*=4), 4 days of G-CSF (*n*=3) or 4 days of G-CSF+1 dose of BOP (*n*=3). Data are representative of three independent experiments. (**b**) Analysis of PB progenitor and HSC content following a single dose of BOP+AMD3100 (*n*=8), 4 days of G-CSF (*n*=3), 4 days of G-CSF+1 dose of AMD3100 (*n*=9) or 4 days of G-CSF+1 dose of BOP+AMD3100 (*n*=8). Data are a pool of two independent experiments. (**c**) Schematic of serial competitive transplant assay. (**d**) Proportion of PB RFP^+^ white blood cells (WBC) in 1° recipients. Each data point is an individual recipient from one of two transplants. 1st=crosses, 2nd=circles. Dashed line is mathematically expected engraftment level. (**e**) Analysis of PB RFP^+^ and GFP^+^ lymphoid (B220^+^ and CD3^+^) and myeloid (Gr1^+^/Mac1^+^) engraftment in 1° recipients. *n*=8; pooled from 1st and 2nd experiments. (**f**) 2° recipient PB analysis. Each data point is an individual recipient. Dashed line is mathematically expected engraftment level. (**g**) Analysis of PB RFP^+^ and GFP^+^ lymphoid (B220^+^ and CD3^+^) and myeloid (Gr1^+^/Mac1^+^) engraftment in 2° recipients 20 weeks post transplant. Data are grouped based on the 5 individual 2° donors. *n*=4 2°recipients per donor. UD=undetectable. Around 7–8-week-old female C57 mice were used for **a** and **b**. Around 6–7-week-old male RFP and GFP and 9-week-old female C57 recipients were used for **d** and **e**. Secondary C57 recipients were 7 weeks old (**f**,**g**). All data are mean±s.e.m. Analysis using one-way ANOVA (**a**,**b**), or *t*-test (**d**,**f**) *P<0.05, **P<0.01, ***P<0.005, ****P<0.001.

Multiple reports have shown G-CSF-dependent mobilization can be synergistically augmented by co-treatment with cytotoxic agents, inhibitors of CXCR4 and inhibitors of α_4,_ α_L_ and β_1_ (refs 9, 18, 40, 41, 42). Consistent with these reports, the treatment of mice with a single dose of BOP following 4 days of G-CSF resulted in significant synergistic increases in the incidence and the total numbers of progenitors (LSK) and HSC (LSKSLAM cells) relative to G-CSF alone (Supplementary Fig. 3a,b and Fig. 4a, respectively).

Importantly, in comparison to mobilization with 4 days of G-CSF, equivalent numbers of progenitors and equivalent proportion and number of HSC were mobilized using a single dose of the combination of BOP and AMD3100 (Fig. 4b and Supplementary Fig. 3c,d, respectively). In contrast, the combination of G-CSF and AMD3100 mobilized significantly more progenitors and HSC (Fig. 4b; Supplementary Fig. 3c,d). However, the greatest number of progenitors and HSC were mobilized with the combination of 4 days of G-CSF followed by a single dose of AMD3100 and BOP with a significant synergistic increase compared with G-CSF alone or the combination of AMD3100 and BOP (Fig. 4b).

To compare the hematopoietic potential of PB mobilized with multiple doses of G-CSF versus a single dose of BOP and AMD3100, a competitive long-term reconstitution assay was used (Fig. 4c). Despite equivalent numbers of HSC being mobilized following G-CSF and the combination of BOP and AMD3100 (Fig. 4b), significantly enhanced short and long-term multi-lineage engraftment was observed in the PB and BM using the latter (Fig. 4d,e and Supplementary Fig. 3e,f, respectively). Furthermore, this significantly greater engraftment than was mathematically expected was maintained in the PB and BM of secondary transplants (Fig. 4f,g and Supplementary Fig. 3g,h, respectively). The greater engraftment observed with BOP and AMD3100-mobilized PB suggests cells with the LSK/LSKSLAM phenotype mobilized by G-CSF have reduced hematopoietic potential, which is consistent with previous findings demonstrating G-CSF-mobilized LSK cells have significantly impaired engraftment potential relative to native BM LSK^43^.

### BOP+AMD3100 mobilizes human CD34^+^ HSC in humanized mice

**Figure 5:**
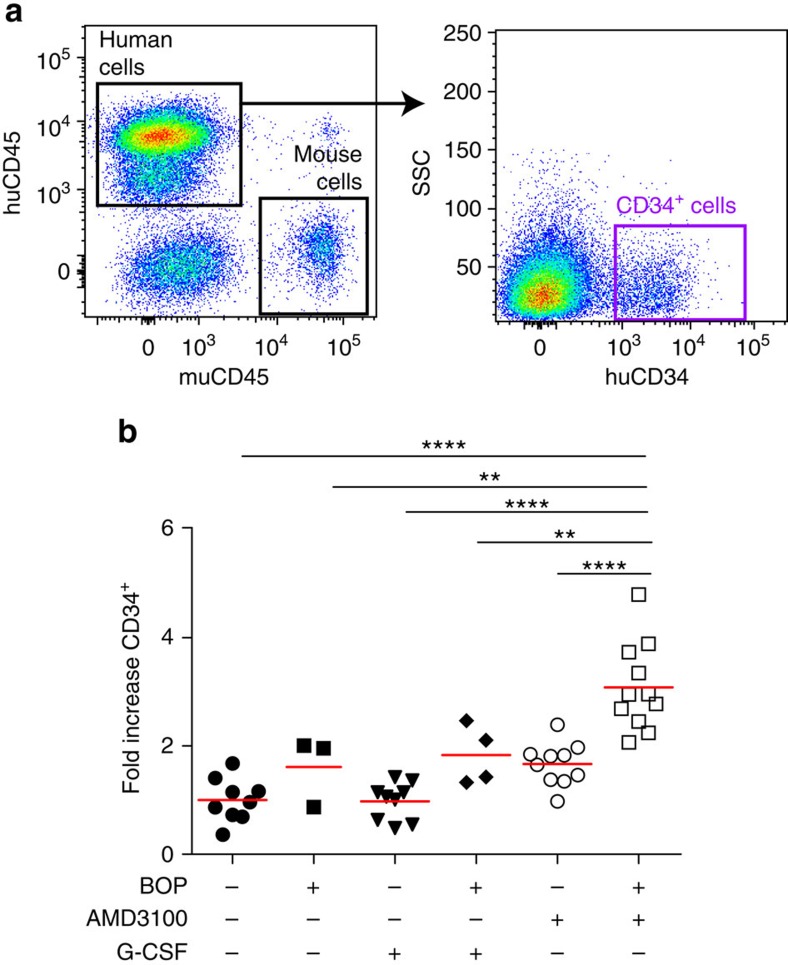
The combination of BOP and AMD3100 effectively mobilizes human CD34^+^ stem and progenitors in humanized NSG (huNSG) mice. (**a**) Representative flow cytometric plot of huNSG PB. (**b**) Analysis of huCD45^+^CD34^+^ cell content in huNSG PB following mobilization. Data are expressed as fold increase in CD34^+^ cells per ml PB relative to saline control and each data point represents an individual animal. Red bar, mean. CB CD34^+^ cells were transplanted into 6–10-week-old female and male NSG mice. Analysis using one-way ANOVA, **P<0.01, ****P<0.001.

To determine whether HSC mobilization using BOP is equivalent in humans, we utilized huNSG mice (Fig. 5a)^24^,^44^. Treatment with a single dose of BOP or AMD3100, or multiple doses of G-CSF for 4 days alone did not result in a significant increase in PB human WBC (Supplementary Fig. 4) or human CD34^+^ stem and progenitors (Fig. 5b). In contrast, a single dose of BOP in combination with AMD3100 resulted in a significant increase in both human WBC (Supplementary Fig. 4) and stem and progenitors (Fig. 5b). These data demonstrate huNSG mice are a useful surrogate model for human HSC mobilization and demonstrate the promising efficacy of BOP and AMD3100 for rapid clinical mobilization of human CD34^+^ cells.

## Discussion

Mobilization of HSC and progenitors for PB stem cell transplants has been clinically adopted for over 30 years. In normal healthy donors, HSC mobilization is used in over three-quarters of allogeneic transplants and is predominantly achieved using standard multi-dose G-CSF over several days. In recent times, significant advances have been made to address the shortcomings of G-CSF (reviewed in ref. 45) such as the development of CXCR4 antagonists including the FDA-approved AMD3100 (refs 8, 9) and experimental agents such as ALT-1188 (ref. 38) and TG-0054 (ref. 46). For mobilizing healthy donors, identifying novel agents that effectively, safely, rapidly and selectively mobilize HSC in a single dose remains clinically important.

HSC are known to express a large number of integrins including α_4_β_1_ and α_9_β_1_, which have critical roles in HSC regulation in BM^16^. Previous studies have confirmed that the inhibition of integrin α_4_β_1_ and VCAM-1 interactions using neutralizing antibodies or specific small molecule inhibitors effectively mobilize HSC in both mice and primates^6^,^18^,^19^. However, the role of the related α_9_β_1_ in this context is unexplored, in large part due to the lack of effective anti-α_9_-blocking antibodies that target HSC *in vivo* or specific α_9_-targeting small molecules. However, given the role α_4_β_1_ plays in HSC mobilization and the fact that α_9_ and α_4_ share several ligands including VCAM-1 and trOpn, a role for α_9_β_1_ in HSC mobilization would not be unexpected. Previously, *in vitro* studies using α_9_-blocking antibodies were found to inhibit CD34^+^ cell proliferation, differentiation and adhesion to primary osteoblasts^47^. In addition, we demonstrated α_9_β_1_/trOpn interactions are potently chemotactic for HSC and blocking antibodies directed to α_9_β_1_ completely inhibits the migration of HSC *in vitro*^16^. α_9_β_1_ has also been reported to mediate G-CSF receptor signalling pathways and contribute to granulopoiesis, as mice lacking α_9_ have reduced BM granulocyte precursors as well as impaired neutrophil development in response to G-CSF^48^.

In this study, we demonstrate targeting of α_9_β_1_/α_4_β_1_ using a single dose of BOP^22^, induces rapid mobilization of long-term repopulating HSC through inhibiting integrin-dependent binding, most likely to trOpn^15^,^16^ and VCAM-1 (ref. 17). Using the fluorescent BOP analogue, R-BC154, we demonstrate a significant proportion of binding to human and murine HSC occurs through α_9_β_1_, whereas binding to lineage-committed cells is almost exclusively through α_4_β_1_. Consequently, inhibition of α_9_β_1_ was found to provide an added advantage to HSC mobilization yields over inhibition of α_4_β_1_ alone as demonstrated by the significantly greater mobilization of HSC and progenitors using BOP in comparison to the selective α_4_β_1_ antagonist BIO5192; particularly when used in combination with AMD3100. The reduced synergism in HSC mobilization, but not WBC mobilization using BIO5192 in combination with AMD3100 compared with BOP in combination with AMD3100 supports targeting α_4_ biases towards progenitor mobilization whereas targeting α_9_ preferentially mobilizes HSC. The pronounced increase in AMD3100-mediated mobilization using BOP relative to BIO5192 suggests the role of integrin α_9_β_1_ in HSC mobilization is magnified with concomitant targeting of CXCR4.

As expected, with the distinct mechanisms of action between BOP, AMD3100 and G-CSF, the combination of these three agents resulted in the greatest increase in LSK and LSKSLAM mobilization when compared with any other combination. Thus, BOP in combination with G-CSF and AMD3100 is likely to have significant advantages over mobilization using the combination of G-CSF and AMD3100; although further research is required to define the functional capacity of these mobilized blood products. Currently, the combination of G-CSF and AMD3100 is most commonly used in autologous transplants, as the combination is effective and superior to G-CSF alone^49^. However, in a clinical context, the use of AMD3100 has advantages compared with G-CSF, as it directly targets HSC, without altering the function of the HSC niche, or bone formation^3^, which is especially important for the mobilization of normal donors. As a consequence, the use of BOP, which also directly targets HSC, will be most advantageous in combination with AMD3100 as opposed to BOP alone, as a potential rapid, single dose alternative to G-CSF for the specific targeting of HSC in the mobilization of normal donors for allogeneic transplants.

We have previously shown BM HSC residing within the endosteal region exhibit greater hematopoietic potential than HSC isolated from the central medullary region^14^,^29^. The functional characteristics of the endosteal BM have been a topic of great interest since the concept of a stem cell niche was originally postulated by Schofield^50^. Studies highlighted the endosteal niche as a hypoxic environment^51^ where cells such as osteoblasts^52^,^53^, endothelial cells^54^, perivascular cells^12^, megakaryocytes^55^ as well as their associated ECM proteins are important regulators of HSC maintenance and function. Herein, we show HSC and progenitors isolated from the endosteal region express α_9_β_1_/α_4_β_1_ that are in a higher affinity, ligand-primed binding conformation relative to cells from the central compartment. Although the physiological relevance of this differential integrin activity remains unknown, these observations are consistent with previous reports that HSC-dependent binding between trOpn and α_4_β_1_ and α_9_β_1_ is restricted to endosteal BM^15^,^16^.

One possible explanation for the enhanced priming/activation of integrins on HSC in endosteal BM are their close proximity to bone. Bone has a very high mineral content and is the primary storage site of the inorganic salts of calcium and magnesium as well as trace metals such as manganese^56^,^57^: all of which are known to play a role in α_9_β_1_ and α_4_β_1_ adopting the conformational changes required for ligand binding^21^,^22^,^58^. Using XFM and quantitative ICP-MS, we demonstrate that endosteal BM harbours a significantly greater concentration of elemental calcium, magnesium and manganese relative to the central medullary region. The data strongly suggest this high divalent metal ion content plays a previously unrecognized role in differential integrin priming/activation within BM. Although direct evidence of divalent metal concentration gradients in BM have not been reported until now, the concept has previously been invoked to rationalize the preferential localization of HSC within endosteal BM via recognition of extracellular Ca^2+^ through the G protein-coupled calcium-sensing receptor^59^. Collectively, these observations further define the unique nature of the endosteal niche, the differential influence it confers in seemingly phenotypically identical cells and provides validation for therapeutic targeting of the endosteal stem cell niche for stem cell therapies^60^,^61^.

G-CSF induces the mobilization of BM HSC and progenitors by causing a ‘cytokine storm' that results in the cleavage of key retentive HSC interactions such as CXCR4/SDF-1 (refs 1, 2) and VCAM-1/α_4_β_1_. In addition, evidence supports G-CSF and/or chemotherapy treatment causing a reduction in the homing and engraftment potential of mobilized PB compared with BM HSC, which has been suggested to be due to disruption of the HSC microenvironment^62^. In addition, previous studies in mice showed G-CSF-mobilized PB LSK cells have reduced colony-forming capacity and long-term reconstitution potential compared with their untreated BM counterparts^43^,^63^. Furthermore, in healthy human donors mobilized using G-CSF, apheresis products contained reduced frequencies of NOD/SCID repopulating cells compared with AMD3100-mobilized PB from the same donors^64^. The current study adds to these findings, demonstrating G-CSF mobilizes proportionally more phenotypic LSK progenitors than LSKSLAM HSC and following a transplant of equal volumes of mobilized PB, the use of G-CSF resulted in a reduced capacity to sustain long-term hematopoiesis relative to mobilized PB using the combination of BOP and AMD3100. Together, these results support previous findings that G-CSF-mobilized HSC and progenitors have reduced *in vitro* and *in vivo* capacity relative to their well-defined BM counterparts in steady state^43^.

Variability observed with HSC mobilization following G-CSF is well documented, both within patients and in different mouse strains^65^. For example, BALB/c mice are effectively mobilized by G-CSF, whereas C57BL/6 mice are considered ‘poor mobilizers'^39^. Although the NODSCIDIL2Rγ^−/−^ mouse model has routinely and successfully been used to assess the hematopoietic potential of human HSC and progenitors^24^, previous studies have not assessed the ability of G-CSF to effectively mobilize CD34^+^ cells in humanized NODSCIDIL2Rγ^−/−^ mice. The current data demonstrate significant impairment. In contrast, the combination of BOP and AMD3100 resulted in significant mobilization of human CD34^+^ cells, suggesting the use of this strategy may be effective in clinical mobilization of human donors.

In summary, we demonstrate that a single dose of BOP, a small molecule targeting α_4_β_1_ and α_9_β_1_ integrins, effectively and rapidly mobilizes HSC with long-term multi-lineage engraftment potential, identifying a previously unrecognized role for α_9_β_1_ in HSC mobilization. When used in combination with CXCR4 inhibitors such as AMD3100, significantly enhanced mobilization of long-term repopulating HSC was observed relative to G-CSF. The efficacy of HSC mobilization using the BOP and AMD3100 combination was recapitulated in the mobilization of CD34^+^ cells in humanized NODSCIDIL2Rγ^−/−^ mice. Using the related fluorescently labelled integrin antagonist R-BC154, we show that this class of compounds bind murine and human HSC and progenitors via endogenously activated/primed α_4_β_1_ and α_9_β_1_ within the endosteal niche. Furthermore, we show that the majority of R-BC154/BOP binding to human HSC occurs via α_9_β_1_, which is absent on lineage-committed cells. These results highlight an effective and convenient strategy of therapeutically targeting endosteal HSC which address many of the shortcomings associated with G-CSF and paves the way for the development of additional selective small molecule α_9_β_1_ integrin antagonists for preferential HSC mobilization.

## Methods

### Compounds and reagents

R-BC154 and BOP were synthesized according to previously published methods^21^,^22^. BIO5192 (Tocris Bioscience), AMD3100 (octahydrochloride hydrate salt) (Sigma-Aldrich) and human G-CSF (Filgrastim, Amgen, Thousand Oaks, CA, USA) were used without further purification. For *in vivo* experiments, lyophilized powders of R-BC154 and BOP sodium salts were dissolved in 10% hydroxypropyl-β-cyclodextrin (HPβCD) in saline (w/v). A stock solution of BIO5192 in DMSO (10 mg ml^−1^) was diluted to 0.1 mg ml^−1^ in 10% HPβCD/saline immediately before use.

### Mice

C57BL/6, red fluorescent protein (RFP), green fluorescent protein (GFP), α_4_^flox/flox^α_9_^flox/flox^ vav-cre, NODSCIDIL2Rγ^−/−^ (NSG) mice were bred at Monash Animal Services (Monash University, Clayton, Australia). Conditional α_4_^flox/flox^α_9_^flox/flox^ mice were initially generated by cross-breeding α_4_^flox/flox^ mice with α_9_^flox/flox^ mice and vav-cre mice. For transplants, irradiation was given in a split dose (5.25 Gy each) 6 h apart, 24 h before transplant for C57BL/6 mice and in a single dose (2.75 Gy) 5 h before transplant for NSG mice and a total of 2 × 10^5^ irradiated (15 Gy) C57BL/6 BM cells or 2 × 10^6^ irradiated (15 Gy) umbilical CB MNC given as support cells to each recipient, respectively. All experiments were approved by Monash Animal Services Ethics Committee.

### Umbilical CB and human BM

Umbilical CB and human BM were obtained from the Mercy Hospital for Women (East Melbourne, Australia) and patients undergoing hip replacement at St Vincent's Public Hospital or St Vincent's Private Hospital (East Melbourne, Australia), respectively^15^. All experiments were following informed consent and approved by the ethics committee of each respective hospital and written consent was obtained from all subjects.

### Human CD34^+^ cell isolation

Low-density MNC were isolated from human CB or BM by discontinuous density centrifugation using Ficoll-Hypaque (1.077 g ml^−1^, Pharmacia Biotech, Sweden). Lineage-negative cells were then isolated using mouse-anti-human CD3, CD11b, CD14, CD16, CD20, CD24 and CD235a (BD, Franklin Lakes, NJ, USA), and Dynal sheep anti-mouse immunoglobulin G (IgG) beads (Invitrogen, Carlsbad, CA, USA). Cells were then stained with CD34-FITC and CD38-PE (BD, Franklin Lakes, NJ, USA) for subsequent fluorescence-activated cell separation^15^,^16^. All antibodies were used at optimal pre-titred concentrations (Supplementary Table 2).

### Murine hematopoietic cell isolation

For the isolation of murine central marrow, the ends of femurs, tibias and iliac crests were removed and the bones flushed using a 21- or 23-gauge needle. For the isolation of endosteal cells, the marrow-depleted bones and the bone ends were ground in a mortar and pestle. The bone fragments were washed, incubated in 3 mg ml^−1^ Collagenase I (Roche, Basel, Switzerland) and 4 mg ml^−1^ Dispase II (Worthington, Lakewood, NJ), in phosphate-buffered saline (PBS; 310 mOsm) in a shaking incubator (37 °C; 250 r.p.m. for 5 min), then washed by vigorously shaking^66^. RBC in collected PB were lysed using NH_4_Cl lysis buffer for 5 min at room temperature.

### Generation of humanized NSG mice

Humanized NSG mice were generated by transplanting sorted CB CD34^+^ cells (1.5–2.0 × 10^5^) with 2 × 10^6^ irradiated (15 Gy) CB MNC. After 4 weeks, PB was assessed for the proportion of human CD45^+^ cells using anti-huCD45 and muCD45 antibodies.

### Cell lines

LN18 and Chinese hamster ovary (CHO) cells were obtained from the ATCC. α_4_β_1_ and α_9_β_1_ expressing human LN18 cells were maintained in DMEM supplemented with 2 mM L-glutamine and 10% fetal bovine serum (FBS)^21^. CHO cells overexpressing murine α_9_ and α_4_ were generated by retroviral transduction using pMSCV-mα_9_-IRES-eGFP and pMSCV-mα_4_-IRES-eGFP^16^, and maintained in DMEM/F-12 medium supplemented with 2 mM L-glutamine and 10% FBS.

### Flow cytometry

Flow cytometric analysis was performed using an LSR II (BD Biosciences)^16^. R-BC154 was detected at either 582 or 610 nm and excited with the yellow-green laser (561 nm). For BM and PB analysis cells were analysed at 10–20,000 cells per s. Cell sorting was performed on a Cytopeia Influx (BD)^16^. All sorts were re-analysed with a purity of >98%.

### Antibody cocktails

For analysis of human WBC from CB MNC or BM and PB from huNSG mice, cells were processed as described above and immunolabelled with anti-huCD34 and anti-huCD38 antibodies. For analysis of huNSG mice, anti-huCD45 and anti-muCD45 antibodies were also included. For analysis of murine progenitor cells (LSK; Lineage^−^Sca-1^+^c-kit^+^) and HSC (LSKSLAM; LSKCD150^+^CD48^−^), BM and PB cells were immunolabelled with a lineage cocktail (anti-B220, anti-CD3, anti-Gr-1, anti-Mac-1), anti-Sca-1, anti-c-kit, anti-CD48 and anti-CD150 antibodies. For lineage analysis, cells were stained with anti-CD3, anti-B220, anti-Mac-1 and anti-Gr-1 antibodies. A full list of antibodies and their working concentrations used in this work is detailed in Supplementary Tables 1 and 2.

### Expression of α_4_ and α_9_β_1_ integrins on human HSC

The expression of human α_4_ (CD49d) and α_9_β_1_ on human HSC from CD34^+^ enriched human BM cells, BM from huNSG mice and CB MNC CD34^+^ cells were assessed by the sequential labelling of cells with purified mouse-anti-human α_9_β_1_, goat-anti-mouse-AF647 followed by a cocktail of mouse-anti-huCD49d-PECy7, anti-huCD34-FITC and anti-huCD38-BV421. Matching mouse IgG1 isotypes were used as controls.

### *In vitro* and *in vivo* R-BC154 binding

For *in vitro* labelling experiments, up to 5 × 10^6^ BM cells from C57 mice, conditional α_4_^−/−^/α_9_^−/−^ vav-cre mice, huNSG, CB MNC and sorted CD34^+^ cells, were treated with R-BC154 (up to 300 nM) in PBS (0.5% BSA) or TBS (0.5% BSA) containing no exogenous cations or 1 mM CaCl_2_/MgCl_2_. *In vitro* labelling experiments were not conducted using Mn^2+^ in the binding buffer as BIO5192 has previously been shown to exhibit low levels of binding to α_9_β_1_ under these conditions^23^. R-BC154 staining in the presence of 10 mM EDTA was used as the negative control. For *in vivo* experiments, C57BL/6 and huNSG mice received subcutaneous injections of R-BC154 (5–10 mg kg^−1^) at 100 μl per 10 gm mouse weight and PB and BM analysed as described above. For competitive displacement of *in situ*-bound R-BC154, WBM cells were treated with 500 nM BOP in PBS containing 0.5% BSA and 1 mM CaCl_2_/MgCl_2_ for 45 min at 37 °C before flow cytometric analysis.

### Determination of α_9_β_1_ contribution to R-BC154 binding

For the assessment of α_9_β_1_ contribution to R-BC154 binding, human and murine cells were stained with R-BC154 (100 nM) in the absence of competing antagonist or in the presence of excess amounts of the selective α_4_β_1_ antagonist BIO5192 (1 μM) or dual α_4_β_1_/α_9_β_1_ antagonist BOP (1 μM) in PBS containing 0.5% BSA and 1 mM CaCl_2_/MgCl_2_ at 0 °C for 30 min. Cells were washed, immunolabelled as described above and analysed by flow cytometry. The contribution of α_9_β_1_ binding was calculated by measuring the difference in MFI between R-BC154+BIO5192 and R-BC154+BOP and expressed as a % of R-BC154 alone. Transduced LN18 cells and CHO cells incubated under identical conditions were used as controls, which confirmed BIO5192 inhibited >95% of R-BC154 binding to α_4_β_1_ but did not inhibit binding to α_9_β_1_. In contrast, BOP inhibited >95% of R-BC154 binding to both α_4_β_1_ and α_9_β_1_. For single-cell correlation analysis, scaled fluorescence intensity values for both R-BC154 binding to α_9_β_1_ in the presence of BIO5192 (to prevent any binding to α_4_β_1_) and α_9_β_1_ parameters were determined for individual CD34^+^CD38^−^ cells using FlowJo X. Linear regression analysis was performed using GraphPad Prism.

### Measurement of calcium gradients in BM

*Sample preparation*. C57BL/6 mice were perfusion fixed with 4% PFA, femurs excised and infiltrated and embedded in methacrylate as per the manufacturer's instructions (JB-4 Plus embedding kit, ProSciTech, Australia). Sections (15 μm) of diaphyseal BM were cut using a tungsten carbide knife and transferred onto 10 × 10 mm silicon nitride (Si) windows (4 mm^2^ window size and 2 μm thickness; Melbourne Centre for Nanofabrication, Australia). Sections were dried and stored in a dehumidified environment before XFM analysis.

*X-ray fluorescence microscopy*. Specimens were analysed using the XFM beamline at the Australian Synchrotron^67^. Unlike full-field imaging common in optical microscopy, XFM is a scanning probe technique where the sample is translated through a focused beam of incident radiation. A beam of 12.7 keV X-rays was focused to a spot of 2.5 μm (full-width at half-maximum of the intensity) using a Kirkpatrick-Baez mirror pair. Using these settings, the intensity profile at the focal plane is a Gaussian distribution^68^ with a s.d. of 1.06 μm. The proportion of incident light which would illuminate from bone at a distance of 20 μm is determined as ∼10^−81^, excluding any possible signal contamination from the high levels of bone Ca. Nevertheless, analysis of calcium content directly at the bone/BM interface (∼0–20 μm from bone; shaded area in Fig. 2h) was avoided and quantitation of calcium was restricted to >20 μm from the bone surface. We have previously arbitrarily defined the interface between endosteal and central BM as ∼12 cell diameters (or ∼100 μm) from the bone surface^13^.

The X-ray energy was chosen to induce K-shell ionization of elements with atomic numbers below 34 (Z<Se). The specimen was continuously scanned through the focus using a virtual pixel size of 2 μm, well matched to the beam profile. Full x-ray fluorescence (XRF) spectral images were obtained at each pixel using an effective dwell time of 31.25 ms. The low-latency, large solid angle 384-channel Maia XRF detector was positioned in the back-scatter geometry and the resulting elemental maps ranged up to 142,129 pixels in size. Quantification using the Maia system is based on a fundamental parameter approach able to operate in real time and has no requirement for explicit per-element calibration^69^,^70^. Two single element foils, Mn and Pt (Micromatter, Canada), were scanned during the experiment and provided sufficient information for use as experimental references to establish elemental quantitation. Deconvolution of the X-ray fluorescence spectra was performed using GeoPIXE v6.3 (ref. 69; CSIRO, Australia) and calibrated using the metal foil measurements. Images were analysed using ImageJ and the relative calcium content quantified by measuring the pixel intensities from lines of interest (*n*=10) extending from the endosteum towards the central vein.

### ICP-MS analysis of BM calcium, magnesium and manganese

Femurs, tibias and iliac crests (3 mice pooled per group, *n*=3) were excised, the epi- and metaphyseal regions removed and the bones flushed with a total of 1.2 ml of cation-free PBS to obtain central BM cellular fractions. The endosteal cellular fraction was harvested by gently grinding the flushed bones, and epi- and metaphyseal fragments in a small mortar and pestle using a total of 1.2 ml of PBS. This level of grinding has previously been demonstrated not to impact cell viability or function^14^,^29^.

The extracellular fractions were collected in the supernatant following cell centrifugation. To control for the grinding ‘selectively contaminating' the eBM samples, bones were flushed in PBS to remove central marrow cells, then flushed and incubated in 3 mg per ml collagenase I and 4 mg per ml dispase II to release the residual-bound cells and proteins as per routine endosteal marrow isolation procedure, followed by further flushing with PBS. The empty bones were then vigorously ground in 0.5 ml PBS and the released cation concentrations were measured. This grinding was significantly more intensive than that which is normally used to release endosteal cells.

To separate the cell-associated cations into membrane-associated and intracellular fractions, half of the cell pellets were treated with 10 mM EDTA to remove any membrane-associated cations. All cell pellets were then frozen at −80 °C, freeze dried and digested with 50 μl of concentrated nitric acid 65% (Suprapur, Merck) overnight at room temperature followed by heating at 90 °C for 20 min to complete the digestion. The reduced volume after digestion was ∼35 μl and each sample was made up to 1 ml by the addition of 965 μl of 1% (v/v) of nitric acid diluent. The central and endosteal supernatants were centrifuged at 10,000*g* to remove any residual cellular debris and a 100 μl aliquot diluted 10-fold with 1% (v/v) nitric acid diluent. ICP-MS measurements were obtained on an Agilent 7700 series ICP-MS instrument under routine multi-element operating conditions using a Helium Reaction Gas Cell. The instrument was calibrated using 0, 5, 10, 50, 100 and 500 p.p.b. of certified multi- element ICP-MS standard calibration solutions (ICP-MS-CAL2-1, ICP-MS-CAL-3 and ICP-MS-CAL-4, Accustandard) for a range of elements. A certified internal standard solution containing 200 p.p.b. of Yttrium (Y89) was used as an internal control (ICP-MS-IS-MIX1-1, Accustandard). Results were calculated as micrograms of metal per gram of wet weight (μg g^−1^) or μmol l^−1^ for the cellular fraction and extracellular fraction, respectively, and then normalized to micrograms of metal per two hindlimbs or cell number, respectively.

### HSC mobilization

Mice received subcutaneous injections at 100 μl per 10 gm body weight of: a single injection of BOP (up to 15 mg kg^−1^) for various timeframes, a single BIO5192 injection at 1 mg kg^−1^ for 1 h, a single AMD3100 injection at 3 mg kg^−1^ for 1 h (mice also receiving BOP or BIO5192 were injected with a single dose of BOP or BIO5192 1 h before harvest), or G-CSF at 250 μg kg^−1^ twice daily (500 μg kg^−1^ day^−1^), 6–8 h apart for 4 consecutive days (mice also receiving BOP and/or AMD3100 were injected with a single dose of BOP and/or AMD3100 1 h before harvest). Control mice received an equivalent volume of saline or 10% HPβCD/saline where appropriate.

### Long-term transplant assays

*Limiting dilution transplants*. RFP mice were treated with either BOP, AMD3100 or a combination of BOP and AMD3100 and PB harvested after 1 h. Equal volumes of PB from each donor from each treatment group was pooled, red cells lysed and WBC resuspended in 200 μl PBS per recipient. Irradiated WBM filler cells were added and then resuspended in PBS for transplantation by tail vein injection. Multi-lineage RFP engraftment was assessed at 6, 12 and 20 weeks post transplant.

*Competitive limiting dilution transplants*. RFP and GFP mice were treated with BOP+AMD3100 and G-CSF, respectively, before PB was harvested and equivalent blood volumes pooled within each treatment group. Red cells were lysed and equal volumes (based on original pre-lysed blood volume) of RFP and GFP PB were transplanted with irradiated WBM filler cells in 200 μl per recipient. RFP and GFP engraftment assessed after 6, 12 and 20 weeks. After 20 weeks WBM cells (1/10th of a femur) from individual primary recipients (*n*=5) were transplanted into irradiated C57 secondary recipients (*n*=4 per primary recipient) and assessed for multi-lineage engraftment after 6, 12 and 20 weeks. Greater than 1% B220^+^, CD3^+^ and Gr1Mac1^+^ donor engraftment was considered multi-lineage.

### Statistical analysis

Data were analysed using unpaired or paired student's *t*-test or one-way analysis of variance where appropriate. If the difference between group means was statistically significant, *post hoc* analysis using the Tukey approach was performed. For determination of stem cell repopulation frequency, Poisson analysis using L-CALC software (Stem Cell Technologies) was used. A log-rank (Mantel–Cox) test was used to compare survival curves. *P*<0.05 was considered significant.

## Additional information

**How to cite this article:** Cao, B. *et al.* Therapeutic targeting and rapid mobilization of endosteal HSC using a small molecule integrin antagonist. *Nat. Commun.* 7:11007 doi: 10.1038/ncomms11007 (2016).

## Supplementary Material

Supplementary Information Supplementary Figures 1-4 and Supplementary Tables 1-2
