# Supplementary material for: Therapeutic targeting and rapid mobilization of endosteal HSC using a small molecule integrin antagonist
Source: Nat Commun. 2016 Mar 15;7:11007. doi: 10.1038/ncomms11007 (PMC4796355; doi:10.1038/ncomms11007)
Supplement: Supplementary Information — Supplementary Figures 1-4 and Supplementary Tables 1-2 [file ncomms11007-s1.pdf]

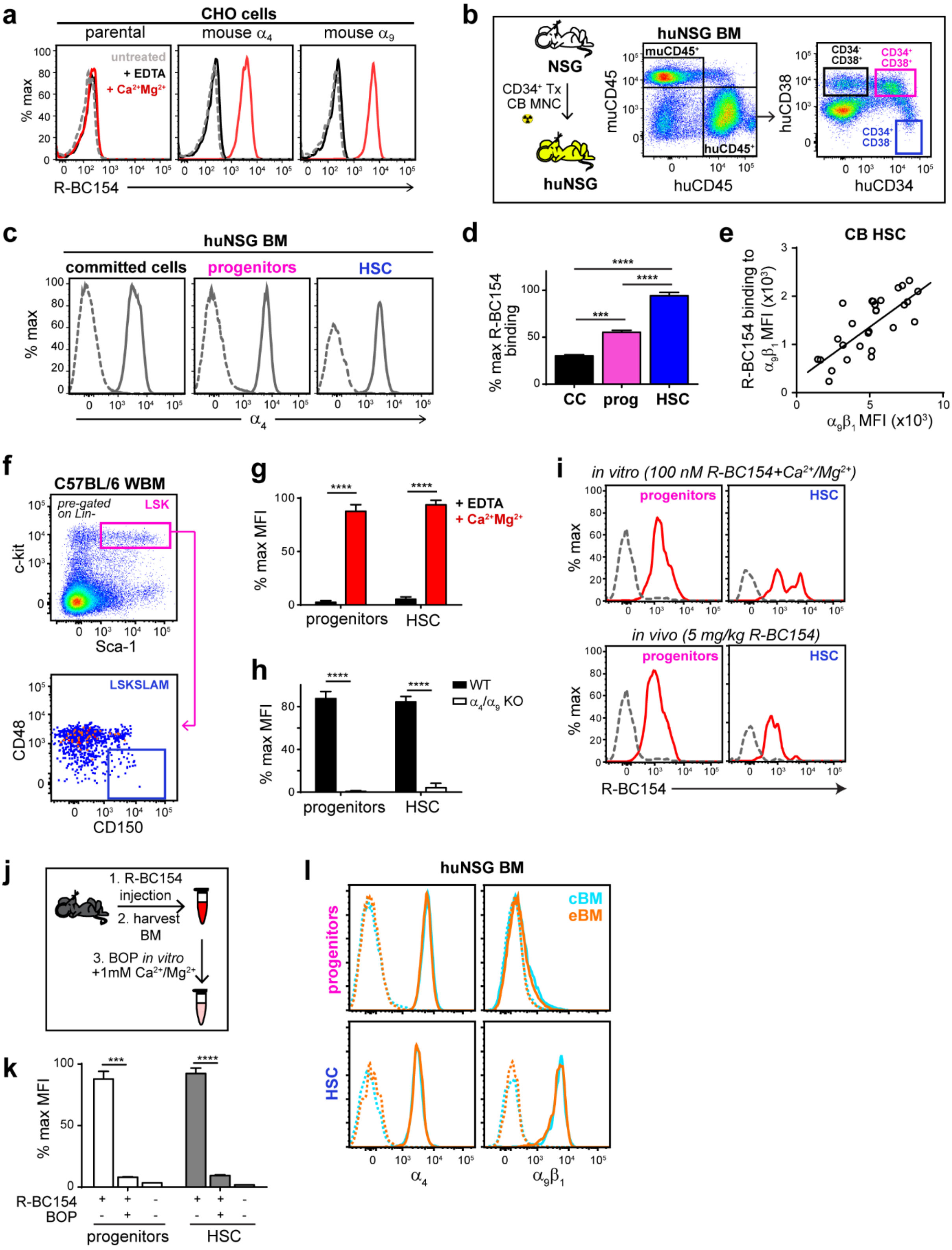

a
CHO cells
parental
mouse α4
mouse α9
untreated
+ EDTA
+ Ca2+Mg2+
% max
R-BC154
b
NSG
CD34+ Tx
CB MNC
huNSG
huNSG BM
muCD45+
huCD45+
muCD45
huCD45
CD34- CD38+
CD34+ CD38+
CD34+ CD38-
huCD38
huCD34
c
huNSG BM
committed cells
progenitors
HSC
% max
α4
d
% max R-BC154 binding
CC
prog
HSC
e
CB HSC
R-BC154 binding to α9β1 MFI (x10³)
α9β1 MFI (x10³)
f
C57BL/6 WBM
pre-gated on Lin-
LSK
c-kit
Sca-1
LSKSLAM
CD48
CD150
g
% max MFI
+ EDTA
+ Ca2+Mg2+
progenitors
HSC
h
% max MFI
WT
α4/α9 KO
progenitors
HSC
i
in vitro (100 nM R-BC154+Ca2+/Mg2+)
progenitors
HSC
% max
in vivo (5 mg/kg R-BC154)
R-BC154
j
1. R-BC154 injection
2. harvest BM
3. BOP in vitro +1mM Ca2+/Mg2+
k
% max MFI
R-BC154
BOP
progenitors
HSC
l
huNSG BM
cBM
eBM
progenitors
HSC
α4
α9β1

Supplementary Figure 1.
(a) R-BC154 specifically binds murine α_4 and α_9 expressed by CHO cells in the presence of divalent metal cations (Ca^{2+}/Mg^{2+}) (red line). R-BC154 binding is abrogated in the presence of EDTA (black line). **(b)** Generation of humanized NODSCIDIL2R$\gamma^{-/-}$ (huNSG) mice from cord blood (CB) mononuclear cells (MNC) and flow cytometric analysis of BM white blood cell (WBC) $CD34^{+}CD38^{+}$ progenitors, $CD34^{+}CD38^{+}$ HSC and $CD34^{-}CD38^{+}$ committed cells. **(c)** Expression of α_4 (solid line) on huNSG BM committed cells, progenitors and HSC. Isotype = dashed line. **(d)** Quantified data from Fig. 1k depicting R-BC154 binding in the presence of Ca^{2+}/Mg^{2+} to huNSG BM committed cells (CC), progenitors (prog) and HSC. n=3. **(e)** Positive correlation of $\alpha_9\beta_1$ expression versus R-BC154 binding to $\alpha_9\beta_1$ (P<0.0001). *P*-value represents statistically significant non-zero slope using linear regression analysis. Each circle depicts a single CB $CD34^{+}CD38^{-}$ cell and data is representative of 3 individual CB samples. **(f)** Dot plot of murine BM progenitors ($Lin^{-}Sca\text{-}1^{+}c\text{-}kit^{+}$; LSK) and HSC ($LSKCD150^{+}CD48^{-}$; LSKSLAM). **(g)** R-BC154 binding to murine progenitors and HSC in the presence of Ca^{2+}/Mg^{2+} (red bar) or EDTA (black bar). n=3. Representative of at least 3 independent experiments. **(h)** R-BC154 binding in the presence of Ca^{2+}/Mg^{2+} to WT and α_4 and α_9 knockout (KO) BM murine progenitors and HSC. n=3. **(i)** Representative comparison of *in vitro* staining of R-BC154 in the presence of exogenous Ca^{2+}/Mg^{2+} and *in vivo* administration of R-BC154. n=3. **(j)** Schematic of assessment of the specificity and reversibility of R-BC154 binding *in vivo*. **(k)** Displacement of R-BC154 binding to BM progenitors and HSC *in situ* using exogenous competing BOP *in vitro*. n=3. **(l)** Expression of α_4 and $\alpha_9\beta_1$ (solid line, dashed line IgG1 isotype controls) on central (cBM) and endosteal (eBM) progenitors and HSC from huNSG BM. 7-8 week old female NSG mice were transplanted with CB $CD34^{+}$ cells (**b**,**c**,**d**,**l**). 7-8 week old female mice were used for (**f**,**g**). 6-12 week old female and male mice were used for (**h**). 8 week old female mice were used for (**i**-**k**). All data is mean±s.e.m. Analysis using one-way ANOVA (**d**,**k**) or student's t-test (**g**,**h**), ****P*<0.005, *****P*<0.001.

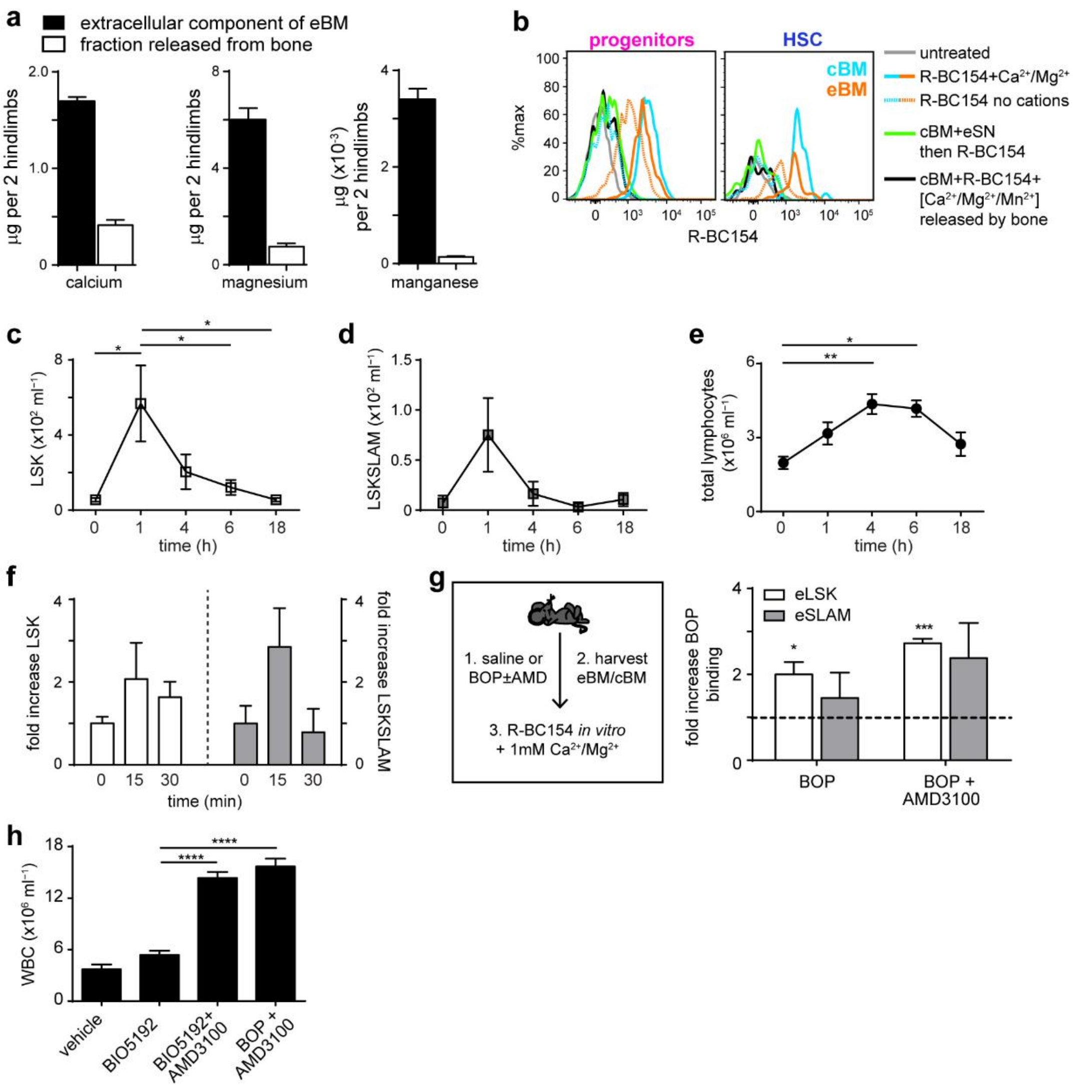

Supplementary Figure 2.

(a) Total calcium, magnesium and manganese content in the extracellular component of endosteal BM (eBM) compared to the amount mechanically released from bone during endosteal cell isolation. **(b)** Analysis of R-BC154 binding to cBM progenitors (LSK) and HSC (LSKSLAM) when pre-treated with eBM supernatant (eSN) or with exogenous $Ca^{2+}/Mg^{2+}/Mn^{2+}$ at concentrations determined in (**a**). R-BC154 binding to eBM and cBM LSK and LSKSLAM in the presence of 1 mM Ca^{2+}/Mg^{2+} or in the absence of cations were used as controls. Data is representative of 4 samples. **(c)** Extended time-course analysis of total LSK, **(d)** LSKSLAM and **(e)** lymphocyte content in PB for up to 18 hours after a single dose of BOP. n=5. **(f)** Analysis of LSK and LSKSLAM content in PB of mice treated with saline or R-BC154 over 30 minutes. Data is a pool of 2 independent experiments. n>6. **(g)** Analysis of *in vivo* BOP binding to endosteal versus central LSK and LSKSLAM following the administration of BOP alone or BOP in combination with AMD3100. Data is presented as fold increase in BOP binding to endosteal cells relative to their central counterparts (dotted line). n=3. *P*-values represent statistically significant results using paired t-test between central and endosteal populations within each treatment group. **(h)** PB white blood cell (WBC) content 1 hour after a single dose of vehicle (n=5), BIO5192 alone (n=5) or in combination with AMD3100 (n=5), or BOP in combination with AMD3100 (n=3). 7-8 week old female mice were used for (**a-g**). 7-8 week old male mice were used for (**h**). All data is mean±s.e.m. Analysis using one-way ANOVA (**c**,**d**,**e**,**f**,**h**), **P*<0.05, ***P*<0.01, ****P*<0.005, *****P*<0.001.

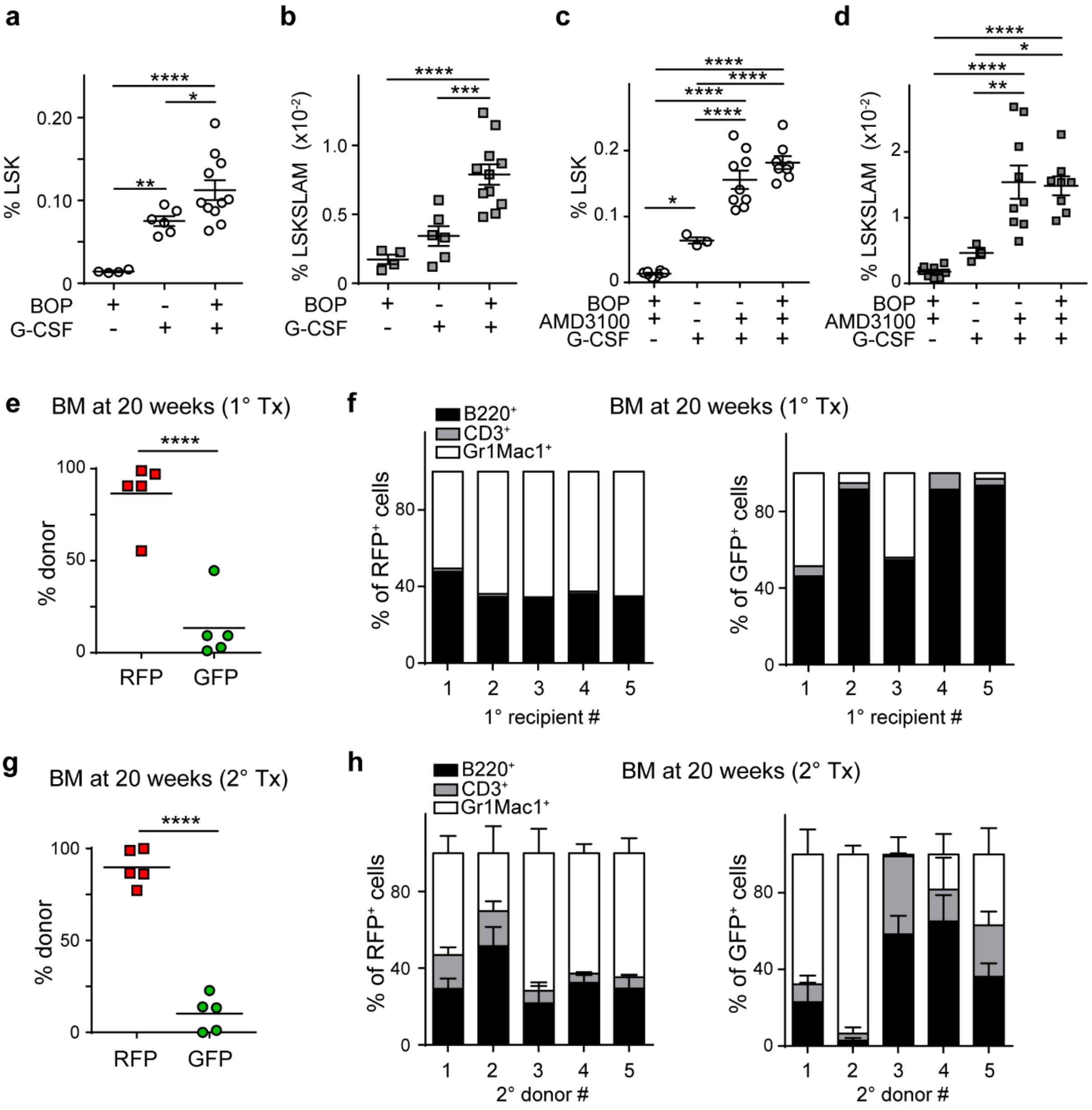

Supplementary Figure 3.

Analysis of the proportion of **(a)** PB progenitors (LSK) and **(b)** HSC (LSKSLAM) following a single dose of BOP (n=4), 4 days of G-CSF (n=6) or 4 days of G-CSF + 1 dose of BOP (n=11). Data is a pool of 3 independent experiments. Analysis of the proportion of **(c)** PB progenitors (LSK) and **(d)** HSC (LSKSLAM) following BOP+AMD3100 (n=8), 4 days of G-CSF (n=3), 4 days of G-CSF + 1 dose of AMD3100 (n=9) or 4 days of G-CSF + 1 dose of AMD3100+BOP (n=8). Data is a pool of 2 independent experiments. **(e)** Analysis of donor engraftment and **(f)** lineage distribution (lymphoid, $B220^+$ and $CD3^+$ and myeloid, $Gr1^+$/$Mac1^+$) in BM of 1° recipients 20 weeks post-transplant. **(g)** Analysis of donor engraftment and **(h)** lineage distribution (lymphoid, $B220^+$ and $CD3^+$ and myeloid, $Gr1^+$/$Mac1^+$) in BM of 2° recipients 20 weeks post-transplant. All symbols are individual animals and all data is mean±s.e.m. 7-8 week old female C57 mice were used for (**a-d**). 6-7 week old male RFP and GFP donors and 9 week old female C57 recipients were used for (**e**,**f**). Secondary C57 recipients were 7 weeks old (**g**,**h**). Analysis using one-way ANOVA (**a**,**b**,**c**,**d**) or student's t-test (**e**,**g**), *P<0.05, **P<0.01, ***P<0.005, ****P<0.001.

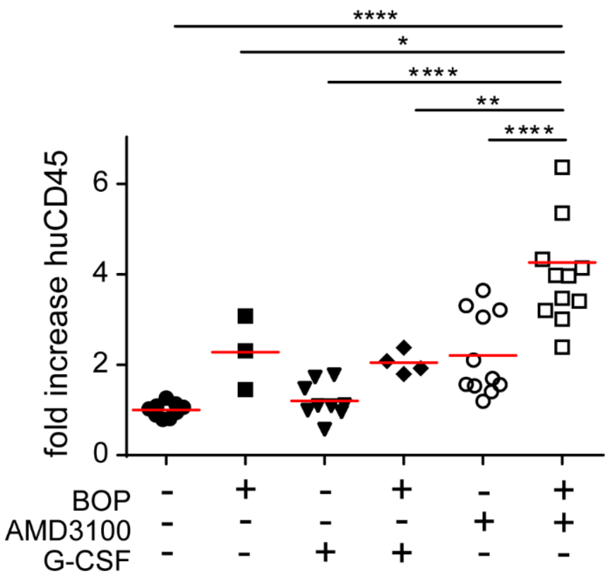

Supplementary Figure 4.
Analysis of PB human WBC in huNSG mice treated with saline, 1 dose of BOP, 4 days of G-CSF, 4 days of G-CSF + 1 dose of BOP, 1 dose of AMD3100 and 1 dose of AMD3100+BOP. Each data point is an individual animal and the red bar represents the mean (n=3 to 11) pooled from 3 independent experiments. CB $CD34^+$ cells were transplanted into 6-10 week old female and male NSG mice. Analysis using one-way ANOVA, *P<0.05, **P<0.01, ****P<0.001.

Supplementary Table 1. Anti-mouse antibodies

Antibody	Clone	Isotype	Cat #	Concentration
**CD3e**	17A2	rat IgG2b	100234	1 μg ml^{-1}
**CD4, L3T4**	GK1.5	rat IgG2b	553729	1 μg ml^{-1}
**CD8a, Ly-2**	53-6.7	rat IgG2a	553031	1 μg ml^{-1}
**CD11b, Mac-1**	M1/70	rat IgG2b	101218	0.25 μg ml^{-1}
**CD45**	30-F11	rat IgG2b	564279	1 μg ml^{-1}
**CD45R, B220**	RA3_6B2	rat IgG2a	563103<br>103226	1 μg ml^{-1}<br>0.5 μg ml^{-1}
**CD48**	HM48-1	A. hamster IgG1	103404	1 μg ml^{-1}
**CD117, c-kit**	2B8	rat IgG2b	564011	1 μg ml^{-1}
**CD150 (SLAMF1)**	TC15-12F12.2	rat IgG2a	115931	1 μg ml^{-1}
**GR-1, Ly-6G**	RB6-8C5	rat IgG2b	108418	0.25 μg ml^{-1}
**Sca-1,Ly-6A/E**	E13-161.7	rat IgG2a	122514	0.4 μg ml^{-1}
**TER119**	TER119	rat IgG2b	560509	0.4 μg ml^{-1}

Supplementary Table 2. Anti-human antibodies

Antibody	Clone	Isotype	Cat #	Concentration
**CD3**	UCHT1	Mouse IgG1	555330	5 μg ml^{-1}
**CD11b**	ICRF44	Mouse IgG1	555386	5 μg ml^{-1}
**CD14**	M5E2	Mouse IgG2a	555396	5 μg ml^{-1}
**CD16**	3G8	Mouse IgG1	555404	5 μg ml^{-1}
**CD20**	2H7	Mouse IgG2b	555621	5 μg ml^{-1}
**CD24**	ML5	Mouse IgG2a	555426	5 μg ml^{-1}
**CD235a**	GA-R2	Mouse IgG2b	555569	5 μg ml^{-1}
**CD34**	8G12	Mouse IgG1	348053	5 μg ml^{-1}
**CD38**	HIT2	Mouse IgG1	562444<br>347687	2.5 μl per test<br>0.6 μg ml^{-1}
**CD45**	HI30	Mouse IgG1	563717	0.5 μl per test
**CD49d (α_4)**	9F10	Mouse IgG1	304314	1 μl per test
**$\alpha_9\beta_1$**	Y9A2	Mouse IgG1	MAB2078Z	20 μg ml^{-1}
